# Revising PTEN in the Era of Immunotherapy: New Perspectives for an Old Story

**DOI:** 10.3390/cancers11101525

**Published:** 2019-10-10

**Authors:** Geny Piro, Carmine Carbone, Luisa Carbognin, Sara Pilotto, Chiara Ciccarese, Roberto Iacovelli, Michele Milella, Emilio Bria, Giampaolo Tortora

**Affiliations:** 1 Comprehensive Cancer Center, Fondazione Policlinico Universitario Agostino Gemelli IRCCS, 00168 Roma, Italy; 2 Department of Medicine, University of Verona, 37134 Verona, Italy; 3 Division of Gynecologic Oncology, Department of Woman and Child Health and Public Health, Fondazione Policlinico Universitario Agostino Gemelli IRCCS, 00168 Roma, Italy; 4 Medical Oncology, Azienda Ospedaliera Universitaria Integrata, 37134 Verona, Italy; 5 Medical Oncology, Università Cattolica Del Sacro Cuore, 00168 Roma, Italy

**Keywords:** PTEN, immunotherapy, regulatory T cells, immunosuppression

## Abstract

Immunotherapy has emerged as the new therapeutic frontier of cancer treatment, showing enormous survival benefits in multiple tumor diseases. Although undeniable success has been observed in clinical trials, not all patients respond to treatment. Different concurrent conditions can attenuate or completely abrogate the usefulness of immunotherapy due to the activation of several escape mechanisms. Indeed, the tumor microenvironment has an almost full immunosuppressive profile, creating an obstacle to therapeutic treatment. Phosphatase and tensin homolog deleted on chromosome 10 (PTEN) governs a plethora of cellular processes, including maintenance of genomic stability, cell survival/apoptosis, migration, and metabolism. The repertoire of PTEN functions has recently been expanded to include regulation of the tumor microenvironment and immune system, leading to a drastic reevaluation of the canonical paradigm of PTEN action with new potential implications for immunotherapy-based approaches. Understanding the implication of PTEN in cancer immunoediting and immune evasion is crucial to develop new cancer intervention strategies. Recent evidence has shown a double context-dependent role of PTEN in anticancer immunity. Here we summarize the current knowledge of PTEN’s role at a crossroads between tumor and immune compartments, highlighting the most recent findings that are likely to change future clinical practice.

## 1. Introduction

Phosphatase and tensin homolog deleted on chromosome 10 (PTEN) is a major nonredundant, dose-dependent tumor-suppressor gene able to act in both a phosphatidylinositol 3-kinase (PI3K)-dependent and -independent manner [1]. PTEN protein is involved in the regulation of several crucial cell functions, such as maintenance of genomic stability, cell survival, apoptosis, migration, and metabolism, and even partial PTEN loss of function is enough to promote tumorigenesis and accelerate cancer progression [2]. Since its discovery as an elusive tumor suppressor, *PTEN* has been identified as a lost or mutated driver gene in numerous sporadic and heritable tumors [3]. A decade of mechanistic studies has established the intimate role of PTEN and its fine regulation in several animal models and in vitro experiments. In particular, mouse models of main gene mutants and the generation of allelic series in mice with progressively decreased PTEN doses allowed PTEN functional loss to be depicted as a driving force in multiple tumors [2,4,5,6,7,8] and demonstrated that mutated proteins heterodimerize with wild-type PTEN, restraining PTEN activity in a dominant-negative fashion [9]. Consistently, transgenic mouse lines bearing varying levels of wild-type PTEN overexpression acquired anticancer protective features through a healthy metabolism switch, thus opening a route for novel treatment modalities for cancer prevention and therapy [10].

Increasing studies have investigated the potential prognostic and predictive role of PTEN in cancer. However, due to its complex regulation, the mere evaluation of gene mutations is not sufficient to fully uncover the broad range of activity loss status [11]. Indeed, besides genetic alterations, different mechanisms of regulation of PTEN expression and function, including transcriptional regulation, noncoding RNAs, post-translational modifications, and protein–protein interactions, have been reported [12,13]. Interestingly, a new self-regulatory feed-forward loop sustained by PI3K-FOXO-deubiquitinase USP11 in response to PTEN action has been described, which improves its stability and tumor-suppressive activity [14].

Notably, the repertoire of PTEN functions has recently been expanded to include regulation of the tumor microenvironment and immune system, thus changing the canonical paradigm of PTEN action with new potential implications for immunotherapy-based approaches. Here we summarize the current knowledge of the role of PTEN at a crossroads between tumor and immune compartments.

## 2. PTEN Function in Tumor-Immune Microenvironment

Tumor-associated stromal cells, such as fibroblasts and endothelial cells, cooperate with cancer cells to promote proliferation, invasion, and metastasis to distant sites. The immune system orchestrates a primary protective antitumor response; however, tumors often foster a tolerant microenvironment switch, inducing immunosuppressive signals to reduce this protective mechanism.

The basis of tumor-induced anergy has been widely investigated in the last decades, and several studies highlighted the role of T cell unresponsiveness in the early events of tumor progression [15,16,17]. In addition to its cell autonomous effects on cancer cells, PTEN exerts an important regulatory role in tumor microenvironment composition, counteracting the instauration of an immunosuppressive milieu, thus preventing tumor immune escape [18] (Table 1). 

The first evidence of PTEN involvement in immunity emerged in a study correlating its germline deletion with autoimmune disorders [19], followed by a demonstration that T cell-specific PTEN knockout affects T cell homeostasis, inducing spontaneous activation and autoantibody production in mouse models [20].

The emerging literature suggests a central role of PTEN in both innate and adaptive immunity. Myeloid PTEN-deficient mice exhibited augmented collagen deposition, dysregulation of macrophage polarization, and secretion of proinflammatory and profibrotic factors after induction of pulmonary fibrosis [21]. Myeloid-specific depletion of PTEN also increased the recruitment of neutrophils at the inflamed site [22,23]. Conditional deletion of PTEN in B cells led to the acquisition of a hyperproliferative profile [24], while its deletion in dendritic cells caused expansion of CD8^+^ and CD103^+^ subpopulations [25]. PTEN loss in CD4^+^ T cells enhanced their helper function, with augmented activation and production of cytokines [26].

Further studies demonstrated that PTEN controlled cancer cell secretome, avoiding secretion of cytokines, with immunosuppressive potential. In a PTEN-defective melanoma cell line engineered to express PTEN under a tetracycline-responsive promoter, PTEN negatively regulated the expression of the immunosuppressive cytokines interleukin (IL)-10, IL-6, and vascular endothelial growth factor (VEGF) by inhibiting signal transducer and activator of transcription (STAT)3.

The addition of conditioned media from established melanoma cell lines and patient-derived short-term melanoma cultures lacking PTEN to monocyte-derived dendritic cells inhibited the ability of these cells to secrete the inflammatory cytokine IL-12. Blockage of IL-10, IL-6 and VEGF by using neutralizing antibodies partially restored IL-12 production, thus confirming the role of these secreted cytokines in mediating PTEN loss-dependent immunosuppression [27]. An analysis of histological samples from 67 malignant melanoma patients with or without brisk host response showed that PTEN expression was positively correlated with brisk host response [27].

Furthermore, mice bearing PTEN-null senescent prostate tumors displayed a massive tumor infiltration of granulocytic myeloid-derived suppressor cells (MDSCs) recruited in tumor bulk by the unrestricted activation of the Janus kinase (JAK)2/STAT3 pathway and the consequent secretion of chemoattractant molecules. Consistently, treatment with Jak2/Stat3 inhibitors reprogrammed the secreted cytokine network, leading to antitumor immune response and enhanced chemotherapy efficacy [30]. In agreement with these findings, a non-cell-autonomous mechanism has been described, by which prostate cancer driven by PTEN loss may evade senescence stimuli in the early step of tumor progression through tumor microenvironment-derived factors. Indeed, at 7–8 weeks of age, genetically engineered mouse models (GEMMs) with specific deletion of PTEN in prostate epithelial cells (Pten^pc−/−^ mice) developed premalignant prostatic lesions characterized by a strong senescence response that counteracted tumor progression. At this stage, PTEN-null lesions showed a concomitant presence of both senescent and proliferative cells; however, the massive infiltration of MDSCs induced secretion of interleukin-1 receptor antagonist (IL-1RA), which hampered the senescence response, thus sustaining tumor growth [31].

Moreover, in mouse models of pancreatic ductal adenocarcinoma, PTEN loss influenced the frequency of intratumoral neutrophils, monocytes, and regulatory T cells (Tregs) through activation of NFkB and expression of chemoattractant cytokines such as CXCL1, G-CSF, and IL-23 [33].

Other deletions may also cooperate with PTEN loss to modify the immune compartment of the tumor microenvironment. For instance, combined deletion of PTEN and Zbtb7a in prostate tumors promoted tumor progression through MDSC recruitment and NF-κB signaling activation, whereas compound loss of PTEN and p53 were associated with an immunosuppressive phenotype [32]. 

A recent preclinical study on gastric cancer models demonstrated that cancer-secreted exosomes were able to deliver specific PTEN-regulating microRNA (miRNA) to MDSCs, inducing their expansion and activation [40].

Notably, a bidirectional regulation mechanism by which PTEN may suppress pro-oncogenic secretome and stroma immune tolerance has been postulated; on the contrary, stroma cells may secrete miRNA-containing exosomes to target PTEN expression in cancer cells. This epigenetic downregulation occurs only at specific metastatic sites and is strictly linked to the specific local microenvironment. More specifically, it has been demonstrated in both human samples and mouse models that astrocyte-derived exosomes mediate an intercellular transfer of PTEN-miRNAs to brain metastatic tumor cells to simulate transient PTEN loss status, which in turn leads to CC-motif chemokine ligand 2 (CCL2) secretion with recruitment of ionized calcium-binding adapter molecule 1 (IBA1)-expressing myeloid cells, thus further enhancing metastatic outgrowth [34]. The fine balance between pro- and antitumorigenic forces decides the fate of cancer progression/inhibition. Moreover, a stroma-specific PTEN signaling pathway that involves the activation of an Ets2-dependent transcriptional program in fibroblast surrounding tumors and suppresses mammary cancer growth has been reported. Mouse models with specific *PTEN* loss in fibroblast showed extended gene expression reprogramming and massive remodeling of the tumor microenvironment, with increased extracellular matrix (ECM) deposition, innate immune cell infiltration, and increased angiogenesis [35,36].

*PTEN* is frequently mutated in sporadic cancers as well as hereditary tumor predisposition syndromes, such as PTEN hamartoma tumor syndrome (PHTS), which increases the risk of benign and malignant tumors, including thyroid cancer. A recent work demonstrated that co-culture of macrophages with a *PTEN*-deficient thyroid cancer cell line induced a strong proinflammatory phenotype compared to PTEN wild-type ones [37]. Further studies in murine knockout models of PHTS demonstrated that a loss of PTEN function led to deregulation of the immune response with a decreased ability of dendritic cells to prime CD8^+^ T cells, leading to impaired tumor eradication [41].

**Figure 1:**
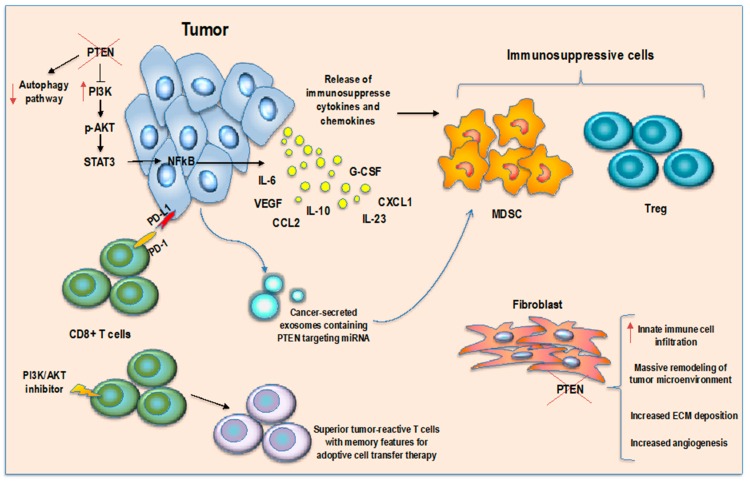
PTEN in cancer immune microenvironment regulation. PTEN loss in tumor cells induces an immunosuppressive microenvironment through secretion of immunosuppressive cytokines and MDSCs/Tregs chemoattractant molecules, inhibition of autophagy pathway and CD8 T cell killing, secretion of miRNA-containing exosomes to target PTEN expression in MDSCs inducing their activation. PTEN loss in fibroblasts surrounding tumor increases immune cell infiltration and induces microenvironment remodeling while inhibition of AKT pathway in T cells enhances memory T cell differentiation.

These findings indicate that PTEN may also have a critical role in immunity, thus demonstrating a functional link between its activities as a tumor suppression driver gene and tumor immune microenvironment regulator gene (Figure 1).

## 3. PTEN Pathway in Regulatory T Cells: A Controversial Role

CD4^+^ FOXP3^+^ regulatory T cells (Tregs) are a fundamental component of the adaptive immune system, able to mediate contact inhibition of effector T cells and deputed to the release of suppressive cytokines such as TGF-β and IL-10 [42]. Cytokine signals drive differentiation of naïve CD4^+^ T cells in Tregs, and several Treg cell functions appear to rely on an intact PTEN/PI3K/AKT pathway [43].

Tumor cells may avoid immune surveillance through upregulation of specific proteins deputed to the maintenance of peripheral immune tolerance, such as programmed cell death ligand-1 (PD-L1), which binds the immune receptor programmed cell death-1 (PD-1) on immune cells. The activated downstream pathway leads to inhibition of cytotoxic T lymphocytes and reduction of tumor-infiltrating T cells; on the other hand, PD-1/PD-L1 interaction induces the differentiation of CD4^+^ T cells into Tregs and enhances their suppressive function [44]. This effect is mediated by the inhibition of AKT phosphorylation and simultaneous PTEN overexpression. 

Tregs increase their regulatory function in response to inflammatory stimuli via induction of PTEN expression [44]. Activated Tregs upregulate PD-1, which in turn increases PTEN phosphatase activity and turnover. This effect is mediated by inhibition of casein kinase 2 (CK2), a serine/threonine protein kinase that regulates PTEN activity [45].

Furthermore, it has been demonstrated that cell contact–dependent potentiation of Treg stability and function is mediated by the interaction between semaphorin-4a, expressed on T effector cells and dendritic cells, and NRP-1, expressed at high levels on Tregs, via activation of the PTEN axis [46].

Indoleamine 2,3-dioxigenase (IDO) is an enzyme involved in immunosuppressive metabolism, catalyzing the rate-limiting step of L-tryptophan (Trp) conversion into L-kynurenine (Kyn) [47]. It exerts strong immunosuppressive effects in physiological conditions such as pregnancy and during tumor progression, affecting both innate and adaptive immunity [48,49,50,51]. Persistent activation of this kynurenine pathway leads to depletion of the Trp pool in immune cells, with a consequential lack of response to immunological stimulus [52]. Moreover, accumulation of Kyn and its derivative products elicits cytotoxic effects on immune effector cells and stimulates Treg differentiation [53,54]. In the past, genetic or pharmacological targeting of the IDO pathway was evaluated as a strategy to counteract tumor spreading [55,56,57]. Interestingly, a permanent PD-1/PTEN-driven feedback loop has been described, in which the immunoregulatory enzyme IDO, expressed on tolerogenic dendritic cells (DCs) and other antigen-presenting cells (APCs), activates the PTEN pathway in Tregs, inducing a reduction of PI3K/AKT activation and thus an increment of FOXO1 and FOXO3a activity, which upregulates PD-1 and PTEN expression, creating an autosustaining persistent PTEN stimulation loop that stabilizes the suppressive Treg population [28,58,59,60]. Thus, IDO and PD-1 work sequentially to sustain the suppressive Treg phenotype. Blockage of PD-1 causes AKT phosphorylation, progressive loss of FOXO3a expression, and abrogation of the suppressive features. Consistently, pharmacological targeting of PTEN after immunotherapy with vaccine/T cells or chemotherapy in melanoma mouse models profoundly reprogrammed the tumor microenvironment from a suppressive to a proinflammatory milieu, with rapid regression of tumors. Moreover, inhibition of PTEN induced an intratumor increment of activated proinflammatory Ly6c^+^CD11b^+^ myeloid dendritic cells, which expressed more CD86 and less PD-L1 [28].

**Figure 2:**
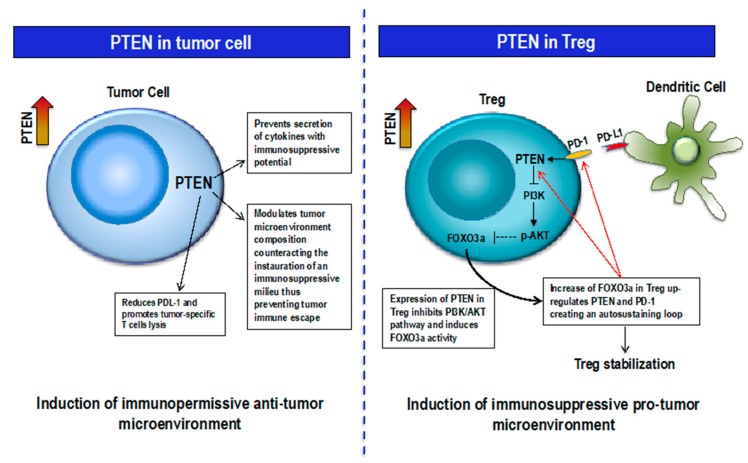
Context-dependent PTEN function. Left, PTEN expression in Tumor cells modulates microenvironment composition with anti-tumor effect; right, PTEN-FOXO3a-PD-1 feedback loop in Treg increases Treg stability thus supporting an immunosuppressive pro-tumor microenvironment.

Therefore, PTEN may act in two opposite ways: as a powerful tumor suppressor if expressed in tumor cells, and as an immune suppressor and tumor escape enhancer if expressed in Tregs (Figure 2).

Genetically modified mice with specific deletion of PTEN in Tregs (PTENTreg-KO mice) showed slow melanoma and Lewis lung carcinoma (LLC) tumor growth and a high grade of inflammation and were not able to create an immunosuppressive tumor microenvironment. Moreover, injection of apoptotic cells in PTENTreg-KO tumor-free mice induced an inflammatory CD11b^+^CD103^+^ myeloid response and elicited no FOXO3a^+^PD-1^+^ Treg recruitment. Similar results were obtained with pharmacological PTEN inhibition [28].

PTEN acts as a negative regulator of IL-2-mediated expansion of Tregs. Indeed, Treg cells lacking PTEN were increased in number [61]. Further studies on PTEN/Treg silenced mouse models demonstrated a propensity of T cells to differentiate into T helper phenotype with an exacerbation of follicular helper and germinal center responses and a global loss of Treg stability. Indeed, PTEN-negative Tregs lost the expression of high-affinity IL-2 receptor (IL-2Rty and FOXP3, and thus their suppressor function [43,62]. Gene-set enrichment analysis showed that the top 10 upregulated genes in PTEN-deficient Tregs were associated with cell cycle pathways. Moreover, ingenuity pathway analysis of differentially expressed genes between wild-type and PTEN-deficient Tregs revealed upregulation of pathways implicated in autoimmune diseases, glycolysis, T helper cell differentiation, and immune signaling in PTEN-deficient cells [62]. 

Overall, these findings demonstrate that PTEN is highly expressed in Tregs and this is essential to retain the characteristic immunosuppressive phenotype. In conflict with these data, it has been shown that mice with a deletion of PTEN in the T cell compartment developed normal Tregs that responded to IL-2 proliferative stimulation while retaining their ability to suppress effector T cells [63]. Moreover, another study reported that specific deletion of the subunit p110o of PI3Ko in Tregs disrupted their function and enabled immune-mediated tumor regression in murine models of lymphoma and breast cancer [64].

## 4. PTEN-Modulating Strategies

### 4.1. Inhibition of PTEN Function

In recent years, PTEN inhibitor drugs have been developed and evaluated for their neuroprotective and proregeneration properties, and subsequently for their anticancer function [65]. The orthovanadate drug VO-OHpic is a high-affinity small-molecule inhibitor of PTEN that has been tested to destabilize the PTEN^+^ Treg population. In melanoma and LLC mouse models, VO-OHpic in combination with low doses of chemotherapy showed a marked synergistic antitumor effect with rapid immune system activation [28,58]. In established tumors, PTEN inhibition did not spontaneously promote tumor regression by itself, but needed inflammatory stimuli induced by chemotherapy or immunotherapy, which sensitized Tregs to PTEN blockade-dependent destabilization. However, besides Tregs, many different cells express PTEN, thus VO-OHpic does not work selectively. Since PTEN acts as tumor-suppressor gene in cancer cells, its wide inhibition can raise some concerns. However, the authors argued that the effects of tumor-suppressor gene blockade occur after a long time, thus one optional strategy would be short-term intermittent treatment, such as pulsed immunotherapy [58].

### 4.2. Reactivation of PTEN Pathway

Regarding the “tumor side” of the problem, recent studies have proposed new interesting strategies to reactivate antitumor PTEN function in tumor cells. For instance, the natural compound indole-3-carbinol has been proposed as a potential therapeutic strategy for cancer prevention and treatment thanks to its induction of PTEN reactivation [66]. Indole-3-carbinol is a natural compound in cruciferous vegetables derived from the breakdown of glucobrassicin [67]. Crystallographic analysis identified a putative indole-3-carbinol binding pocket on WW domain containing E3 ubiquitin protein ligase 1 (WWP1), an enzyme able to suppress dimerization, membrane recruitment, and PTEN function through its K27-linked poly-ubiquitination. In vitro and in vivo studies on prostate cancer models confirmed indole-3-carbinol as a potent WWP1 inhibitor with antitumor activity, thus suggesting this drug for a tumor suppressor reactivation approach [66].

Another proposed strategy to restore functional PTEN is cell-mediated therapy, in which specific cells with tumor-homing ability, such as neural stem cells for glioblastoma, are genetically modified to express a particular protein with therapeutic potential. An alternative PTEN isoform produced by translation from an alternative upstream start codon called PTEN long (PTEN-L) has been described. Unlike conventional PTEN, this isoform can be secreted and then captured by neighboring cells in the intracellular compartment thanks to a specific signal sequence. An engineered version of PTEN-L with leader sequence from human light-chain immunoglobulin G has been proposed to arm neural stem cells. Interestingly, this modified PTEN-L showed enhanced secretion capacity and an increased propensity to be transferred between cells [68]. Finally, nanoparticle-mediated systemic delivery of PTEN mRNA demonstrated significant inhibition of tumor growth in mouse models of PTEN-null prostate cancer [69].

## 5. PTEN Role in Immunotherapy Response

### 5.1. Immunotherapy

Immunotherapy springs from the primary need to revert T cell tolerance toward tumor antigens. Much effort has been invested in target discovery and immunomodulating strategies, and today cancer immunotherapy represents one of the major breakthroughs of cancer treatment.

The landscape of immunotherapy approaches comprises a plethora of targeting strategies, such as tumor-targeting monoclonal antibodies, adoptive cell transfers, oncolytic viruses, cancer vaccines, dendritic cell-based immunotherapies, immunostimulatory cytokines, immunomodulatory monoclonal antibodies, immunosuppressive metabolism inhibitors, pattern recognition receptor agonists, and immunogenic cell death inducers [70]. Among the others, the best and broadest results have been recently achieved with immune checkpoint inhibitors targeting crucial molecules of antitumor T cell response, such as cytotoxic T lymphocyte associated antigen-4 (CTLA-4), PD-1, and PD-L1. The mechanism of action of CTLA-4 typically involves competition with another T cell surface molecule, cluster of designation 28 (CD28), for binding to B7 proteins CD80 and CD86 in order to deactivate T cells [71]. Tregs constitutively express CTLA-4 on cell membranes, and this is believed to be critical for their immunosuppressive properties [72]. Indeed, CTLA-4 on Treg surfaces can capture the ligands CD80 and CD86 from opposing APC cells by a trans-endocytosis process, thus downregulating their expression and ultimately affecting the potency of APC cells to activate other T cells [73,74].

Similarly, the PD-1 blockade restarts an effective antitumor immune response and reverses Treg-mediated suppression of effector T cells. In physiological conditions, PD-1 activation inhibits phosphorylation of TCR downstream molecules, thus reducing TCR signaling after extensive activation. In pathological conditions, constitutive hyperactivation of PD-1 increases the number of exhausted T cells, resulting in altered functionality and poor control of infections and tumors [75,76]. CTLA-4 and PD-1 differ in location. Whereas CTLA-4 is expressed exclusively by T cell compartment, PD-1 is present also on B cells, natural killer cells, and myeloid cells. Its ligand PD-L1 is expressed on leukocytes and nonhematopoietic cells, and in nonlymphoid tissues [77].

Since the first success of the anti-CTLA-4 ipilimumab in metastatic melanoma, several practice-changing clinical studies have established the usefulness of such treatments in many solid tumor types [77,78]. Actually, approved immune-checkpoint inhibitors include ipilimumab; the anti-PD-L1s atezolizumab, avelumab, and durvalumab; and the anti-PD-1s pembrolizumab and nivolumab.

### 5.2. PTEN and Immunotherapy Resistance

The tumor microenvironment has a nearly full immunosilencing profile, creating a main obstacle to immunotherapy treatment combined with immunogenic chemoradiotherapy. Indeed, although a massive release of tumor antigens occurs following cytotoxic treatment, the recognition machinery frequently fails to cross-present them in an immunogenic fashion.

Studies in the literature report a different context-related role of PTEN in immunity according to cell type. Indeed, the PTEN pathway in Tregs drives stabilization of the suppressive immune cell population and tolerance toward apoptotic cells, thus revealing Treg-specific PTEN targeting as a previously unsuspected strategy for tumor treatment. 

Despite its pro-tumor immunosuppressive role in Tregs, specific loss of PTEN in cancer cells has been linked to reduction of antitumor immunity, with induction of PD-L1 expression on glioma cells through a translational regulation mechanism [38]. Similarly, in patient-derived short-term melanoma cultures that either naturally expressed or lacked PTEN gene and in PTEN knockdown/knock-in cells, PD-L1 expression was inversely correlated with PTEN expression, highlighting PD-L1 modulation as an alternative PTEN-dependent mechanism to promote host immune response against cancer [27].

Consistently, activation of the PI3K-AKT-S6K1 pathway induces immune escape and apoptosis of activated T cells, impairing tumor-specific T cell killing through upregulation of PD-L1 on colorectal [79], lung [80], renal [81], breast, and prostate cancer cells [82]. Recent studies highlighted an association between PTEN expression and immunotherapy response (Table 2). 

Interestingly, a metastatic non-small-cell lung cancer (NSCLC) case harboring PTEN mutation, PD-L1 positivity, and high tumor mutation burden showed a lack of durable response to PD-1 inhibitor [87]. An analysis of somatic mutational profiles of 113 NSCLCs treated with immune checkpoint inhibitors revealed that PTEN mutations were only found in nonresponders [86].

Moreover, Peng et al. demonstrated that PTEN knockout in melanoma cells determined protection from cell lysis when co-cultured in vitro with tumor-reactive T cells and decreased T cell trafficking in tumor bulk in adoptive T cell therapy mouse models [29]. More importantly, tumoral PTEN loss in patients correlated with decreased T cell infiltration at tumor sites and poor response to PD-1 inhibitors. In detail, PTEN expression was evaluated in 39 tumor specimens from metastatic melanoma patients treated with pembrolizumab or nivolumab, revealing that high-PTEN tumors (more than 10% cell positivity) displayed significant reduction in tumor size. However, the authors showed no correlation between PTEN and PD-L1 expression either in PTEN knockout cell cultures or PTEN-negative and -positive tumor regions from patient specimens with heterogeneous PTEN expression or in a cohort of 135 resected stage IIIB/C melanoma regional metastases. Thus, the mechanism of tumor protection by PTEN loss does not depend on PD-L1 but is linked to the expression of immunomodulatory cytokines, such as CCL2 or VEGF, and to the impairment of specific autophagy pathways. Indeed, enforced expression of autophagy-related genes in PTEN knockout tumor cells fully restored their susceptibility to T cell killing [29]. Similarly, targeting the PI3K pathway in mouse models with PTEN loss by using a selective small molecule inhibitor improved T cell-induced tumor killing and efficacy of immunotherapy with anti PD-1 and anti-CTLA4 treatments [29].

These findings led to the design of a Phase I/II clinical trial that will test a combination of immunotherapy and targeted therapy with a selective PI3K-beta inhibitor in patients with metastatic melanoma who lack the PTEN gene (NCT03131908).

A recent analysis of whole-exome sequencing data of 110 pretreatment melanoma tumor biopsies with matching germline tissue samples and RNA-seq data from a subset of 42 patients revealed that a lower burden of copy number loss was significantly associated with clinical benefit to CTLA-4 blockade. Among the regions associated with recurrent copy number loss, chromosome 10q emerges as being rich in tumor suppressor genes such as PTEN. In a further analysis, the authors highlighted a closer relationship between PTEN loss and CTLA-4 blockade response with an odds of resistance 5.58 times greater than PTEN proficient tumors [83].

Consistent with these observations, a case study reported that in a patient with metastatic uterine leiomyosarcoma who had an exceptional response to anti-PD-1 pembrolizumab, PTEN was revealed as the principal actor of anti-PD-1 checkpoint blockade response [84]. The authors observed a rapid and marked regression at all tumor sites after four doses of anti PD-1, with the exception of one single nonresponding lesion. The comparative analysis between pretreatment samples and germline tissue revealed biallelic PTEN loss as the possible driver of acquired resistance, exclusively harbored by the resistant metastasis, together with reduced expression of two neoantigens linked to immunoreactivity.

A deconvolution analysis of genome-wide DNA methylation data from the complex cellular mixtures of a wide spectrum of solid cancers demonstrated the existence of “immune hot” and “immune cold” tumors with distinctive prognoses, genomic alterations, cytokine pathway activation, and oncogenic drivers [88]. Moreover, the gene expression signature characteristic of hot tumors has been found in samples from responders to immune-checkpoint inhibitors. Evaluation of the different myeloid cell populations revealed a substantially higher percentage of M1 macrophages and T helper 1 (Th1) cells, together with a better prognosis and higher cytotoxic T lymphocyte response in hot tumors with respect to cold tumors [89]. Contrariwise, cold tumors are enriched with Th2 cells and M2-macrophages, which are associated with poor prognosis and immune-suppressive populations such as MDSCs [90,91]. Of note, copy number analysis showed that deletion of PTEN in melanoma cancer cells often segregates with cold tumors and thus immune depletion and potential resistance to immunotherapy [88]. 

Consistent with these observations, PTEN loss of function seems to be the driver of resistance to anti-PD-1 checkpoint blockade also in glioblastoma [85]. In detail, Zhao et al. fully profiled glioblastoma patients enrolled in a longitudinal study with anti-PD-1 nivolumab or pembrolizumab treatment by whole-exome sequencing, RNA expression, and tissue imaging correlating these features with treatment response. Genomic and transcriptomic analysis in a subset of patients (*n* = 45) showed that PTEN somatic mutations were significantly associated with immunosuppressive expression signatures in non-responder tumors, suggesting that PTEN may play a role in the establishment of a specific tumor-immune microenvironment. Indeed, PTEN mutation was found in 23 out of 32 non-responders and only three out of 13 responders. Conversely, mutations in the mitogen-activated protein kinase (MAPK) pathway are significantly enriched in responders. Of note, the immunosuppressive signature was most associated with the tumor subpopulation expressing the migration marker CD44 in PTEN-mutated tumors rather than Tregs, revealing a central role of PTEN-mutated tumor cells in immune regulation. To further explore the connection between PTEN and immunological features, the authors examined RNA-sequencing data from 172 TCGA samples, demonstrating a significant correlation between PTEN mutation and FOXP3-related transcriptional signature, together with a higher percentage of macrophages, microglia, and neutrophils in the tumor microenvironment. Finally, quantitative multiplex immunofluorescence of matched pre- and post-anti-PD-1 treatment samples showed that PTEN-mutated tumors had a significantly higher level of CD68^+^HLA^−^DR^−^ macrophages, which was previously linked to poor survival in melanoma [92]. In post-treatment specimens, CD3^+^ T cells were enriched only in PTEN wild-type tumor, while PTEN-mutated tumors displayed reduced immune infiltration. The authors speculate that this phenomenon may be due to the changing spatial structure in PTEN-mutant glioblastoma, characterized by increased clustering of tumor cells, which physically hampers immune infiltration. More interestingly, immunosuppression gene sets were elevated in non-responders before immunotherapy, but also in responders following immunotherapy, highlighting the fundamental concept of primary resistance in non-responder tumors, probably led by PTEN mutation, and acquired resistance in initial responders under treatment selection pressure [85]. A still unanswered issue is the possible role of PTEN post-transcriptional/-translational loss of activity in guiding acquired resistance to immunotherapy after initial treatment success.

Another study on primary human glioblastoma cell lines derived from resected patients and co-cultured with matched autologous T cells demonstrated high T cell apoptosis upon contact with PTEN-deficient cancer cells, indicating that PTEN-deficient glioblastoma patients are suboptimal candidates for immunotherapy. Pretreatment of these tumor cell lines with a specific PI3K inhibitor reverses the massive apoptotic effect, suggesting the future exploration of immunotherapy in combination with drugs targeting the PI3K/AKT/mTOR pathway [39]. Similar antitumor effects have been demonstrated after co-treatment with toll-like receptor agonist and PI3K inhibitor [93].

### 5.3. AKT Pathway and Adoptive Cell Transfer Therapy

Adoptive cell transfer therapy (ACT) is a promising cellular immunotherapy approach based on administering ex vivo expanded tumor-specific immune cells to cancer patients. Different cell types can be administered for ACT, such as chimeric antigen receptor (CAR) T cells, TCR transduced T cells, natural killer (NK) cells, and TILs. There are currently two CAR T cell therapies that are approved by the FDA for the treatment of pediatric patients and young adults with refractory or relapsing B cell precursor acute lymphoblastic leukemia and adult patients with refractory or relapsing large B cell lymphoma. Despite encouraging clinical benefits of ACT, a main obstacle for long-lasting therapeutic effects is the insufficient persistence of immune cells after adoptive transfer. Of note, constitutive activation of PI3K impairs immunity response, promoting the development of short-lived terminally differentiated effector T cells at the expense of long-lived memory T cells [94]. Conversely, several studies demonstrated that inhibiting AKT signaling during ex vivo priming promotes features of memory T cells and improves antitumor activity [95,96,97,98,99]. These findings suggest that therapeutic modulation of AKT might be a promising strategy to increase the antitumor immunity of adoptively transferred cells, leading to the design of the first human gene therapy clinical trial to incorporate AKT inhibitor in the T cell manufacturing process (NCT03139370).

## 6. Ongoing Clinical Trials

Clinical trials are currently under way to investigate a potential connection between the PTEN pathway and immunotherapy. An ongoing Phase I study of targeted therapy plus anti-PD-L1 durvalumab in patients with advanced or metastatic solid tumors (MEDIPAC) is evaluating the relationship between mutations in the AKT/PIK3CA/PTEN pathway and response to combination therapy (NCT03772561). Another recent Phase Ib study (not yet recruiting) aims to evaluate the safety and antitumor activity of targeted therapy in combination with durvalumab in patients with advanced solid tumors selected for specific molecular alterations, including PTEN mutation (NCT03842228).

Moreover, several studies are investigating the feasibility and antitumor effect of PI3K-targeting drugs in combination with immunotherapy in patients with metastatic melanoma who lack the PTEN gene (NCT03131908), colorectal cancer (NCT03711058), large B cell lymphoma (NCT03484819), lung cancer (NCT03257722), breast cancer, and gynecologic malignancies (NCT03719326). Currently ongoing clinical trials are schematically summarized in Table 3.

## 7. Conclusions

**Figure 3:**
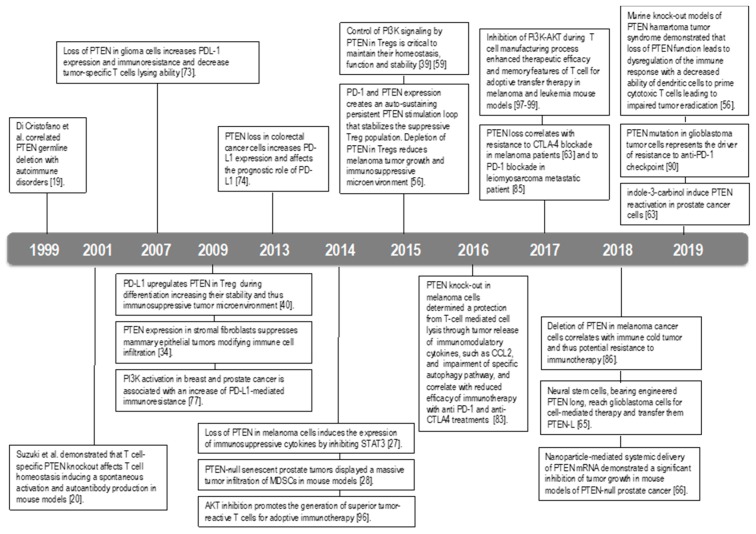
Timeline of the principal findings on PTEN role in immunity.

PTEN is a well-known tumor suppressor gene able to block the proto-oncogenic PI3K/AKT pathway in tumor cells. Notably, the repertoire of PTEN functions has been expanded to include regulation of the tumor microenvironment and the immune system (Figure 3).

Moreover, a controversial function has been reported in Treg-associated PTEN expression with the growing idea of a new paradoxical pro-tumor-like skill. This evidence suggests a double context-dependent role of PTEN according to the specific cell type. Indeed, PTEN expression is crucial to prevent malignant transformation in normal and precancerous cells, and its loss both drives cancer progression and induces a cascade of events that influence the microenvironment toward an immunosuppressive profile. Contrary to its normal role as a tumor suppressor, PTEN-specific expression in Tregs stabilizes these cells, preventing them from switching to an inflamed T helper-like differentiation, thus contributing to the creation of a Treg-dependent immune-suppressive milieu, which finally allows tumor immune escape and survival. Conversely, Treg decrement causes a deep change of tumor microenvironment, with the acquisition of strong immunogenic features that would be suitable for immunotherapy efficacy. A constitutively high level of PTEN protein has been shown in tumor-associated Treg but not in effector T cells. Specifically, it has been demonstrated that Treg cells require PTEN to sustain their suppressive function, supporting the expression of IL-2Rα, a negative modulator of local immune response, and FOXP3 transcription factor. Indeed, mice bearing Treg-specific deletion of PTEN lose Treg homeostasis and stability, gain Th1 response and B cell activation, and are prone to developing autoimmune disease [43,62]. On these bases, the PTEN pathway in Tregs represents an intriguing clinically actionable target to develop novel antitumor immunotherapeutic strategies. 

Given the central role of PTEN in tumor growth and antitumor immune response, interesting approaches have been proposed to target PTEN expression in Tregs or induce its expression in tumor cells. However, the therapeutic potential of each PTEN-modulating approach may be mitigated by the counterpart side effects, raising important concerns. Indeed, systemic administration of PTEN-inhibiting drugs could hit not only PTEN activity in Tregs, but also residual PTEN function in tumor and stroma, with potential pro-tumor consequences. Conversely, enhancing PTEN function could reduce tumor growth while increasing the stability of the immunosuppressive Treg population. A possible strategy to overcome the problems and increase the feasibility of these approaches could be to use more selective modulating strategies or to explore complementary two-step double hit approaches to induce PTEN depletion followed by cell-specific restoration of its pathway. Furthermore, interfering with AKT downstream pathway in adoptively transferred T cells seems to be a promising strategy to promote features of memory T cells and a long-lasting anti-tumor effect.

In recent years, the emerging field of oncoimmunology has fostered an increased understanding of cancer biology, the tumor-immune interrelationship, and the fundamental balance between pro- and antitumor forces. Strong efforts in this direction have guaranteed the development of several immune-modulating strategies able to induce a lasting response in cancer patients. However, different concurrent conditions can attenuate or completely abrogate the usefulness of immunotherapy due to numerous escape mechanisms, such as a lack of immune cell infiltration, poor antigen expression or presentation, tumor-mediated immune suppression, and release of immunomodulatory cytokines. PTEN seems to play a pivotal, context-dependent role in antitumor immune regulation and thus needs to be further investigated.

## Figures and Tables

**Table 1:** Preclinical studies on the role of PTEN pathway in regulation of anti-tumor immunity.

Tumor Type	Tumor Model	Consequence for Immune Regulation and Tumor Immune Microenvironment	Mechanism Involved	Reference
Melanoma	Patient-derived short-term melanoma cultures that either naturally express or lacked PTEN gene; PTEN knock-down/knock-in melanoma cell lines.	Increase of IL-10, IL-6 and VEGF; reduction of secretion of the pro-inflammatory cytokine IL-12 by monocyte-derived dendritic cells.	In melanoma cells lacking PTEN, STAT3 activated the transcription of immunosuppressive cytokines in a PI3K-dependent manner. Moreover, PD-L1 was upregulated, leading to immune evasion.	Dong et al., Oncogene 2014 [27]
Melanoma	Genetically engineered mouse models with specific deletion of PTEN in Tregs (PTENTreg-KO mice).	Intra-tumor increment of activated proinflammatory Ly6c+CD11b+ myeloid dendritic cells, which expressed more CD86 and less PD-L1. Tregs in the tumor lost their suppressive phenotype and converted into proinflammatory helper cells (ex-Tregs).	Genetically modified mice with specific deletion of PTEN in Tregs showed Treg destabilization, slow melanoma tumor growth, high grade of inflammation and were not able to create an immunosuppressive tumor microenvironment.	Sharma et al., Science advances 2015 [28]
Melanoma	Mouse model bearing PTEN deleted melanoma tumors.	Decreased of T cell trafficking in tumor bulk in adoptive T cell therapy mouse models.	Loss of PTEN promoted resistance to T cell killing and decresed T cell infiltration by inducing expression of immunomodulatory cytokines, such as CCL2 or VEGF, and inhibiting autophagy pathway.	Peng et al., Cancer discovery 2016 [29]
Prostate tumor	Mice bearing PTEN-null senescent prostate tumors.	Increase of tumor infiltration of MDSCs. Reduction of CD4+, CD8+ and natural killer (NK) infiltrates.	In PTEN-null senescent tumors, activation of the JAK2/STAT3 pathway via protein tyrosine phosphatase PTPN11/SHP2 established an immunosuppressive tumor microenvironment with production of MDSC chemoattractant cytokines.	Toso et al., Cell reports 2014 [30]
Prostate tumor	Genetically engineered mouse models with specific deletion of PTEN in prostate epithelial cells (Pten^pc−/−^ mice).	Increase of tumor infiltration of MDSCs.	The massive infiltration of MDSCs induced secretion of IL-1 receptor antagonist (IL-1RA) that hampers senescence response thus sustaining tumor growth.	Di Mitri et al., Nature 2014 [31]
Prostate tumor	Genetically engineered mouse models with specific prostate deletion of PTEN (Pten^pc−/−^ mice), PTEN and Zbtb7a (Pten^pc−/−^; Zbtb7a^pc−/−^ mice), PTEN and p53 (Pten^pc−/−^; Trp53^pc−/−^ mice) and organoid cultures.	Increase of tumor infiltration of MDSCs.	Combined deletion of PTEN and Zbtb7a or PTEN and p53 in prostate tumors promoted tumor progression through MDSC recruitment, NF-κB signalling activation and cytokines secretion.	Bezzi et al., Nature medicine 2018 [32]
Pancreatic ductal adenocarcinoma (PDAC)	Genetically engineered mouse models with specific pancreatic deletion of PTEN (Pdx1-Cre, KrasG12D and PtenL mice).	Increase of tumor infiltration of MDSCs, neutrophils, monocytes and Tregs.	PTEN loss induced secretion of chemoattractant cytokines CXCL1, G-CSF, IL-23 via NFkB.	Ying et al., Cancer discovery 2011 [33]
Brain metastatic tumor	Co-culture of tumour cells with primary glia (90% astrocytes). Mouse model obtained by intracarotidly injection of syngeneic mouse melanoma B16BL6 cells to form brain metastase with or whitout astrocyte-specific depletion of PTEN-targeting miRNAs.	Recruitment of ionized calcium-binding adapter molecule 1 (IBA1)-expressing myeloid cells.	Astrocyte-derived exosomes mediated an intercellular transfer of PTEN-miRNAs to brain metastatic tumor cells to simulate transient PTEN loss status which in turn induced secretion of CCL2 with recruitment of IBA1-expressing myeloid cells, thus further enhancing metastasis outgrowth.	Zhang et al., Nature 2015 [34]
Breast cancer	Genetically engineered mouse models with specific inactivation of Pten in stromal fibroblasts of mouse mammary glands.	Massive remodeling of the extra-cellular matrix (ECM), enhanced deposition of collagen, innate immune cell infiltration and increased angiogenesis.	Loss of PTEN in stromal fibroblasts Sustained tumor growth through an Ets2-dependent transcriptional program with induction of MMP9 and CCL3 and VEGF pathway.	Trimboli et al., Nature 2009 [35]
Breast cancer	Genetically engineered mouse models with specific delection of PTEN in fibroblast.	Increase of MMP9, MMP2, BMP1, LOXL2 and EMILIN2, increased angiogenesis.	PTEN loss from mammary stromal fibroblasts activates an oncogenic secretome that orchestrates the transcriptional reprogramming of other cell types in the microenvironment. Downregulation of miR-320 and upregulation of one of its direct targets ETS2, are critical events in Pten-deleted stromal fibroblasts responsible for inducing this oncogenic secretome, which in turn promotes tumour angiogenesis and tumour-cell invasion.	Bronisz et al., Nature cell biology 2011 [36]
Thyroid cancer	Co-culture of PTEN-deficient thyroid cancer cell line with monocytes derived from PTEN hamartoma tumor syndrome (PHTS) patients.	Innate immune cells from PHTS patients acquired a more proinflammatory phenotype and increased lactate production.	Secretion of proinflammatory factors.	Sloot et al., Oncogene 2019 [37]
Glioma	Glioma cell line with genetic deletions in or mutations of PTEN	Increase of immunosuppressive mileu.	Specific loss of PTEN in glioma cells induced reduction of anti-tumor immunity and resistance to tumor-specific T cells lysis with increase of PD-L1 expression through a translational regulation mechanism.	Parsa et al.,Nature medicine 2007 [38]
Glioblastoma	Primary human glioblastoma cell lines derived from resected patients and co-cultured with matched autologous T-cells.	High T-cell apoptosis upon contact with PTEN-deficient cancer cells.	PTEN loss confered immunoresistant phenotype through the PI3K/Akt/mTOR pathway.	Waldron et al., Journal of clinical neuroscience 2010 [39]
Gastric cancer	Mouse models treated with gastric cancer cell derived exosomes.	Increase of MDSCs activation.	Gastric cancer-secreted exosomes were able to deliver miRNA-107 to the host MDSCs inducing their activation through PTEN-downregulation. Indeed, the release of PI3K pathway induced the expression of ARG1 in MDSCs thus increasing their suppressive function.	Ren et al, Cancer Management and Research 2019 [40]

**Table 2:** PTEN pathway and immunotherapy response in patients.

Treatment	Tumor Type	Study Results	n. of Patients	Reference
anti-PD-1 pembrolizumab or nivolumab	Melanoma	Analysis of a cohort of 39 metastatic melanoma patients treated with anti-PD-1 antibodies (pembrolizumab and nivolumab) demonstrated that patients with PTEN positive tumors achieved significantly greater reduction of tumor size than patients with PTEN negative tumors (*p* = 0.029)	Cohort of 39 patients	Peng et al., Cancer discovery 2016 [29]
anti CTLA-4 ipilimumab and/or anti-PD-1 pembrolizumab	Melanoma	Analysis of a cohort of longitudinal tissue samples from metastatic melanoma patients treated with sequential immune checkpoint blockade (CTLA-4 blockade followed by PD-1 blockade at time of progression) demonstrated that PTEN loss is associated with CTLA-4 blockade resistance.	Cohort of 56 patients	Roh et al., Science translational medicine 2017 [83]
anti-PD-1 pembrolizumab	Uterine leiomyosarcoma	Analysis of primary tumor, the sole treatment-resistant metastasis, and germline tissue identified biallelic PTEN loss as potential clinical mechanism of acquired resistance to immune checkpoint therapy.	Case report	George et al., Immunity 2017 [84]
anti PD-1 nivolumab or pembrolizumab	Glioblastoma	Mutations on PTEN were significantly enriched in nonresponders to anti-PD-1 inhibitors. Analysis of matched pre- and post-anti-PD-1 treatment samples showed that PTEN-mutated tumors had a significantly higher level of CD68+HLA-DR− macrophages, which was previously linked to poor survival in melanoma.	Cohort of 76 patients	Zhao et al., Nature medicine 2019 [85]
anti-PD-1 nivolumab and anti-CTLA-4 ipilimumab	Non-small cell lung cancer (NSCLC)	PTEN mutations were significanly associated with resistence to immunocheckpoint inhibitor (*p* < 0.05).	Cohort of 113 patients	Chen et al., Cancer Sci. 2019 [86]
anti PD-1 nivolumab and pembrolizumab	Non-small cell lung cancer (NSCLC)	A metastatic NSCLC case with PTEN mutation, 80% PD-L1 expression and high tumor mutational load showed a durable response to mTORC1 inhibitor but was refractory to treatment with anti-PD-1 antibodies.	Case report	Parikh et al., Lung Cancer 2018 [87]

**Table 3:** Current clinical trials investigating PTEN-PI3K-AKT pathway and immunotherapy.

Conditions	Immunotherapy Treatment	Study Description	State	Estimated Enrolment	Study Identifier
Advanced or metastatic solid tumors	anti PD-L1 durvalumab	This is a Phase I dose-escalation study to evaluate the safety and tolerability of combination treatment of AKT inhibitor AZD5363 + PARP inhibitor olaparib + durvalumab. An exploratory objective is to explore molecular correlates of the relationship between mutations in AKT/PIK3CA/PTEN pathway and treatment response.	Recruiting	40 participants	NCT03772561
Advanced solid tumors selected for specific molecular alterations, including PTEN mutation	anti PD-L1 durvalumab	This is a Phase Ib study to evaluate effects and best dose of the PI3Kinase inhibitior copanlisib and PARP inhibitor olaparib when given together with durvalumab in patients with molecularly-selected solid tumors including PTEN mutation.	Not yet recruiting	102 participants	NCT03842228
Metastatic melanoma with PTEN Loss	anti-PD-1 pembrolizumab	This is a Phase I/II study to evaluate objective response rate and overall survival of the selective PI3K-Beta Inhibitor GSK2636771 in combination with pembrolizumab in patients with metastatic melanoma and PTEN Loss.	Recruiting	41 participants	NCT03131908
Relapsed/refractory mismatch-repair proficient colorectal cancer	anti-PD-1 nivolumab	This is a Phase I/II study to evaluate objective response rate of PI3Kinase inhibitor copanlisib and nivolumab.	Recruiting	54 participants	NCT03711058
Recurrent/refractory diffuse large B-cell lymphoma or primary mediastinal large B-cell lymphoma	anti-PD-1 nivolumab	This is a Phase II study to evaluate objective response rate of PI3Kinase inhibition copanlisib hydrochloride and nivolumab.	Suspended	106 participants	NCT03484819
Metastatic triple-negative breast cancer or ovarian cancer	A2aR and A2bR antagonist AB928	This is a Phase I/Ib study to evaluate safety, tolerability, pharmacokinetic, pharmacodynamic, and clinical activity of immunotherapy combinations. dual adenosine receptor antagonist AB928 in combination with pegylated liposomal doxorubicin with or without PI3kinase-gamma inhibitor IPI-549.	Recruiting	214 participants	NCT03719326
Non Small Cell Lung Cancer	anti-PD-1 pembrolizumab	This is a Phase Ib/II study to evaluate safety and objective response rate of the standard pembrolizumab in combination with the investigational agent PI3K-delta inhibitor idelalisib.	Recruiting	40 participants	NCT03257722
HLA-DPB1*04:01 positive adults with advanced cancers	T Cell Receptor Engineered T Cells (KITE-718)	This is a Phase I study to evaluate safety and objective response rate of KITE-718 treated with AKT inhibitor during manufacturing process.	Recruiting	75 participants	NCT03139370
